# The HIV Pre-exposure Prophylaxis (PrEP) Initiation Cascade: Patient Experiences with Primary Care in a Health Care System Before Starting PrEP

**DOI:** 10.1007/s10461-026-05137-y

**Published:** 2026-04-28

**Authors:** Elizabeth Lockhart, DeAnne Turner, Amjad Alsultan, McKenzie Berezin, Kate Guastaferro, Bianca Pittiglio, Herica Torres Alzate, Laura Szalacha, Stephanie Marhefka

**Affiliations:** 1 Henry Ford Health + Michigan State University, Center for Health Policy and Health Services Research, 1 Ford Place, 5E, Detroit, MI 48202 USA; 2 College of Nursing, University of South Florida, Tampa, FL USA; 3 Department of Social and Behavioral Sciences, New York University, New York, NY USA; 4 Department of Primary Care, Henry Ford Health, Detroit, MI USA; 5 College of Community and Behavioral Sciences, University of South Florida, Tampa, FL USA

**Keywords:** Pre-exposure prophylaxis, HIV, Primary prevention, Retention in care, Primary health care, Prescription drugs

## Abstract

Pre-exposure prophylaxis (PrEP) for HIV prevention has been available since 2012; only 36% of people in the U.S. who would benefit from PrEP have been prescribed it. Primary care is an ideal location to initiate PrEP, as many people who may benefit from PrEP are seen in primary care annually. Yet, patients and providers face barriers to accessing and prescribing PrEP. It is unclear where disengagement from PrEP initiation occurs. We have defined the PrEP Initiation Cascade to include eight timepoints: (1) discussing sexual health with a provider, (2) interest in hearing about PrEP, (3) PrEP awareness, (4) PrEP willingness, (5) PrEP intentions, (6) speaking with a provider about PrEP, (7) being prescribed PrEP, (8) using PrEP. We surveyed patients (n = 278) from primary care in a large health system to understand where they disengaged from initiating PrEP. Multivariable logistic regression models examined patients’ demographic characteristics associated with disengagement during the PrEP Initiation Cascade. Over 66% of patients wanted to hear about PrEP from their provider; less than 20% had. Of patients who spoke to their provider about PrEP, 60% were prescribed it. In regression analyses, we found that patients who were raised religious (vs. never religious), regardless of current religiosity, were more likely to have discussed sexual health with their provider, wanted to hear about PrEP from their provider, and had ever heard of PrEP. People who identified as bisexual (vs. heterosexual) and people who were diagnosed with an STI in the last 6 months (vs. no STI diagnosis) were more likely to have ever spoken to a doctor about PrEP, been prescribed PrEP, and ever used PrEP. Most patients in primary care have discussed their sexual health with their provider in the past year and are interested in hearing about PrEP from their provider, yet few spoke with their provider about PrEP. Multi-level interventions for patients and providers in primary care should be developed to increase PrEP initiation. Future research should focus on keeping patients engaged with the PrEP Initiation Cascade, particularly those in demographic groups shown to disengage in the PrEP Initiation Cascade prematurely.

**Supplementary Information:**

The online version contains supplementary material available at https://doi.org/10.1007/s10461-026-05137-y.

## Introduction

Pre-exposure prophylaxis (PrEP) is an antiviral medication prescribed to prevent human immunodeficiency virus (HIV) acquisition [1]. The U.S. Food and Drug Administration (FDA) has approved two daily oral medications for HIV prevention and two long-acting injectable options. When taken consistently and as prescribed, PrEP is effective at reducing the risk of acquiring HIV via sexual activity by about 99%, and injection drug use by at least 74% [2].

Despite being available for over a decade, PrEP initiation remains low in the U.S.; only 36% of people who could benefit from PrEP have been prescribed it [3], with disparities based on race/ethnicity and sex at birth. As of 2022, 94% of White individuals who could benefit from PrEP have been prescribed it, compared to only 24% of Hispanic/Latino and 13% of Black/African American individuals, while 41% of men who would benefit from PrEP have been prescribed it compared to 15% of women [3]. Low PrEP initiation is attributed to several factors, including suboptimal patient–provider communication and PrEP education [4]. Because PrEP requires a prescription, effective sexual health communication between patients and providers is critical to identifying candidates for PrEP. However, research indicates that these conversations are infrequent across various healthcare settings and populations [5, 6]. Most patients value sexual health discussions and believe providers should initiate them, but report that these conversations do not occur during visits, even when a patient is at elevated HIV risk, such as individuals diagnosed with sexually transmitted infections (STIs) [7, 8].

Beyond discussing sexual health, patients have reported suboptimal levels of PrEP knowledge and awareness. PrEP awareness ranges from 32%—90% among people who are at increased risk of acquiring HIV [9, 10], with these rates increasing over time. Patients may learn about PrEP from many sources, including social media [11], social networks [12], and health care providers, and prefer to hear about PrEP and receive PrEP information from trusted healthcare providers during clinical visits [4].

Providers need to know about behaviors that may put the patient at greater risk of acquiring HIV and can provide PrEP-related information, if they have the requisite knowledge, but face barriers to having these discussions. Competing priorities, time constraints, and insufficient provider training lead to the infrequent collection of sexual history and missed opportunities for engaging in PrEP [4, 13, 14]. Patients report limited communication from their providers about PrEP, highlighting a missed opportunity in clinical settings. For example, a study conducted among PrEP-naïve individuals found that although 85% visited a provider in the past year, only 31% reported discussing PrEP with their provider [15].

**Fig. 1:**
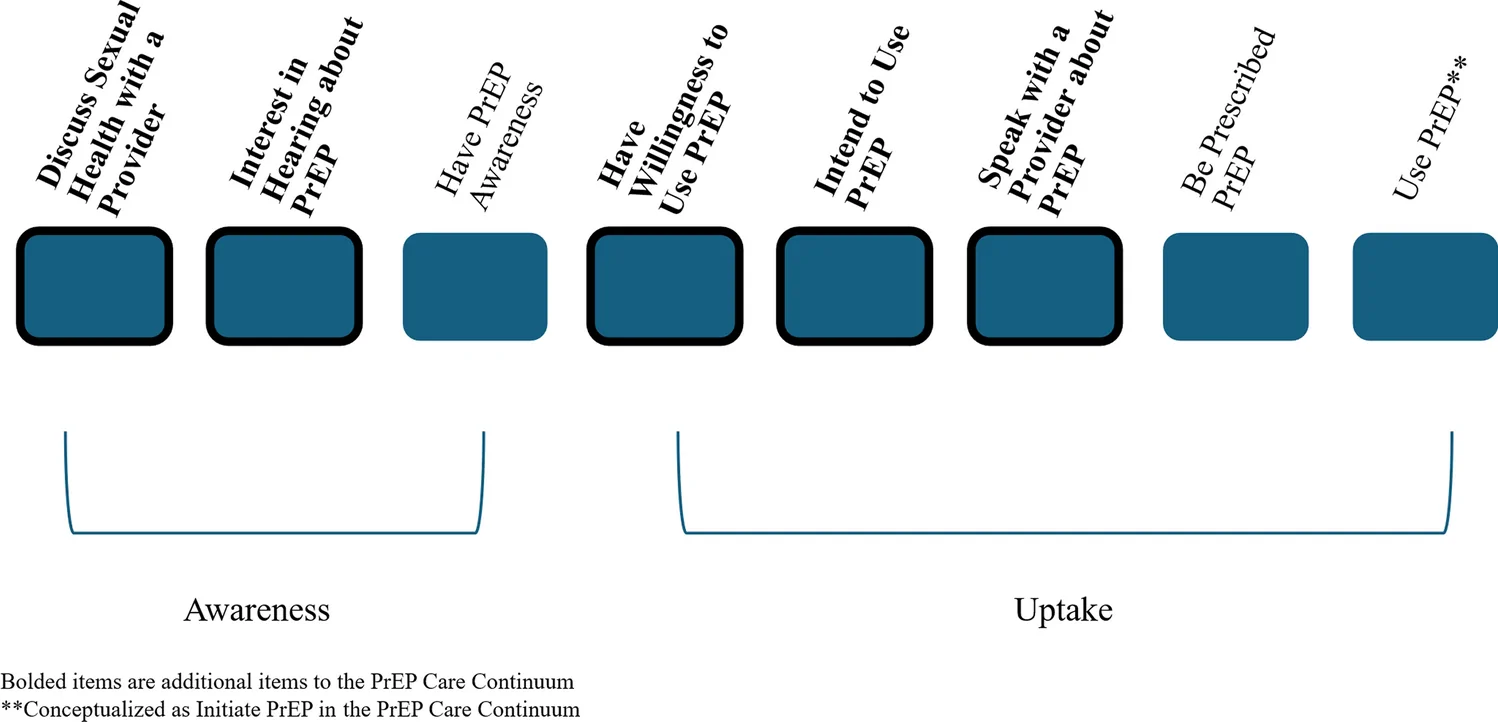
The PrEP Initiation Cascade. Bolded items are additional items to the PrEP Care Continuum. **Conceptualized as initiate PrEP in the PrEP Care Continuum

The PrEP Care Continuum [16] was developed by Nunn and colleagues to define the holistic process of PrEP implementation, including PrEP awareness, uptake, adherence and retention. In the PrEP Care Continuum, PrEP uptake includes facilitating PrEP access, linking to PrEP care, prescribing PrEP, and initiating PrEP. Since PrEP initiation continues to be low, we propose additional factors are necessary prior to PrEP initiation that account for theoretical concepts such as health communication, behavioral willingness and intentions that are not included in the PrEP Care Continuum. The purpose of this study was to identify steps necessary to PrEP initiation, i.e., the PrEP Initiation Cascade (Fig. 1), with a focus on the role of primary care providers (PCPs) and the process by which patients obtain PrEP prescriptions. We sought to understand whether primary care patients discussed their sexual health with their PCPs, if they were aware of PrEP, whether they would like to receive information about PrEP from their provider, if they have spoken to a doctor about PrEP, been prescribed PrEP, and ever used PrEP. Defining the PrEP Initiation Cascade will help identify where individuals disengage from initiating PrEP and ultimately help identify where interventions and support could be provided to assist more people to initiate PrEP.

## Methods

In this cross-sectional study, we sent an anonymous electronic survey to patients at a large learning health system in the U.S. to understand their self-reported experiences with PrEP initiation. Potential participants were aged 18 years or older and attended an internal medicine appointment from December 2023 through April 2024. Most patients in the U.S. visit their primary care provider for both preventive and ongoing care. Quota sampling was used to ensure that feedback was obtained from patients who met and did not meet CDC indications for PrEP (at least 50% of responses were obtained from patients who met indications). CDC indications were matched to International Classification of Disease (ICD-10) codes in the electronic health record indicating possible increased exposure to HIV, including acquisition of an STI (A51, A52, A53, A54, A56, Z20.2, Z20.6); or factors that may put someone at increased risk of HIV acquisition (i.e., “high risk sexual and other behaviors” [Z72.51, Z72.52, Z72.53, W46.1XXA], or “history of sex reassignment” [Z87.890, F64.0, F64.1, F64.2, F64.8, F64.9]). Stratified sampling was used to ensure the race and age distributions of participants were similar to people who were diagnosed with HIV in the same geographic area in 2021 [17]. Surveys were sent to the email associated with the individual’s electronic health record. Participants provided their informed consent electronically and were compensated $30 for completing the survey. This study was approved by the Henry Ford Health Institutional Review Board #16566.

### Measures

#### Dependent Variables

To understand patient experiences regarding PrEP initiation, we asked questions to expand upon the PrEP Care Continuum [16] by including several preparatory steps needed prior to a patient using PrEP. Questions were developed iteratively with clinicians as part of a broader survey seeking patient perspectives on a PrEP uptake intervention. We were interested in current patient experiences with PrEP to understand areas for further intervention. All responses were dichotomized to yes or no/unsure.

*Discussing sexual health with your provider* We asked participants, “In the past 12 months, have you talked with your primary care provider about your sexual health?”

*Interest in hearing about PrEP* We asked participants, “Would you want your provider to tell you about a preventive medication for HIV?”

*PrEP awareness* We asked participants, “Before today, have you ever heard of people taking Pre-Exposure Prophylaxis (PrEP)?”

S*peaking to a doctor about PrEP* We asked participants, “Have you ever spoken to a medical provider about PrEP?”.

*Being prescribed PrEP* We asked participants who indicated that they had ever spoken to a medical provider about PrEP, “Has a medical provider had ever prescribed PrEP for you?”.

*PrEP use* Because prior research has shown that people sometimes take PrEP without a prescription [18], we asked all participants, “Have you ever used HIV pre-exposure prophylaxis, or PrEP, to prevent HIV?”

#### Independent Variables

Participants reported their demographic characteristics, including: age; gender (female, male, transgender female, transgender male, non-binary, another gender); race (Black, White, Asian/Other Pacific Islander, Native American/Alaska Native, Other); sexual orientation (Heterosexual/Straight, Homosexual/Gay/Lesbian, Bisexual, Other); marital status (single/never married, married, widowed, divorced, separated); and education level. We asked participants to report their demographic characteristics, as electronic health record data was incomplete. To have sufficient sample size for analyses, we collapsed response categories. For gender, any participant who indicated any transgender, non-binary, or another gender identity was included in the transgender/non-binary category. For race, any participant who indicated Asian/Other Pacific Islander, Native American/Alaska Native, or Other Race was included in the Another Race/Ethnicity category.

To understand if religious affiliation influenced the PrEP Initiation Cascade, we asked, “*Are you a member of a Church or religious/spiritual institution currently*” and “*Were you a member of a Church or religious/spiritual institution growing up*”? We created a variable that combined these questions so that each participant was designated never religious; raised religious, not currently religious; currently religious not raised religious; or raised and currently religious.

### Statistical Analyses

Descriptive statistics were calculated. T-tests and chi-square tests examined differences between participants who met CDC indications for PrEP and who did not meet CDC indications for PrEP. Multivariable logistic regressions were conducted to determine the association between each outcome and age, gender, race, sexual orientation, marital status, education, and religiosity. All statistical analyses were run using SPSS v.30. Two-sided p-values were used to determine significance (α = 0.05). We report multivariable results as [aOR; (95% CI)].

## Results

A total of 278 patients completed the survey. Slightly more than half of the sample (59%) had not been diagnosed with an STI in the last 6 months. Across the entire sample, participants were, on average, Black (58%), heterosexual/straight (68%), female (45%), and single (68%). See Table 1 and Supplemental Table for full sample description.

**Table 1:** Sample characteristics (n = 278)

Age (M, SD)	35.2 (12.8)
Gender	
Male	112 (40.3)
Female	124 (44.6)
Transgender/non-binary	42 (15.1)
Race	
White	70 (25.2)
Black	161 (57.9)
Another race/ethnicity	47 (16.9)
Sexual orientation	
Heterosexual/straight	190 (68.3)
Homosexual/gay/lesbian	33 (11.9)
Bisexual	32 (11.5)
Other/prefer not to disclose	23 (8.3)
Marital status	
Married	63 (22.7)
Divorced/separated/widowed	27 (9.7)
Single	188 (67.6)
Education	
Less than high school diploma	15 (5.4)
High school diploma	55 (19.8)
Some college but less than a bachelor’s degree	56 (20.1)
Bachelor’s degree	110 (39.6)
Master’s degree or more	42 (15.1)
Religiosity	
Never religious	99 (35.6)
Raised religious, not currently religious	93 (33.5)
Currently religious, not raised religious	13 (4.7)
Raised and currently religious	73 (26.3)
Sexually transmitted infections	
Not diagnosed in last 6 months	165 (59.4)
Diagnosed in last 6 months	113 (40.6)

### Overall Engagement in PrEP Initiation Cascade

**Fig. 2:**
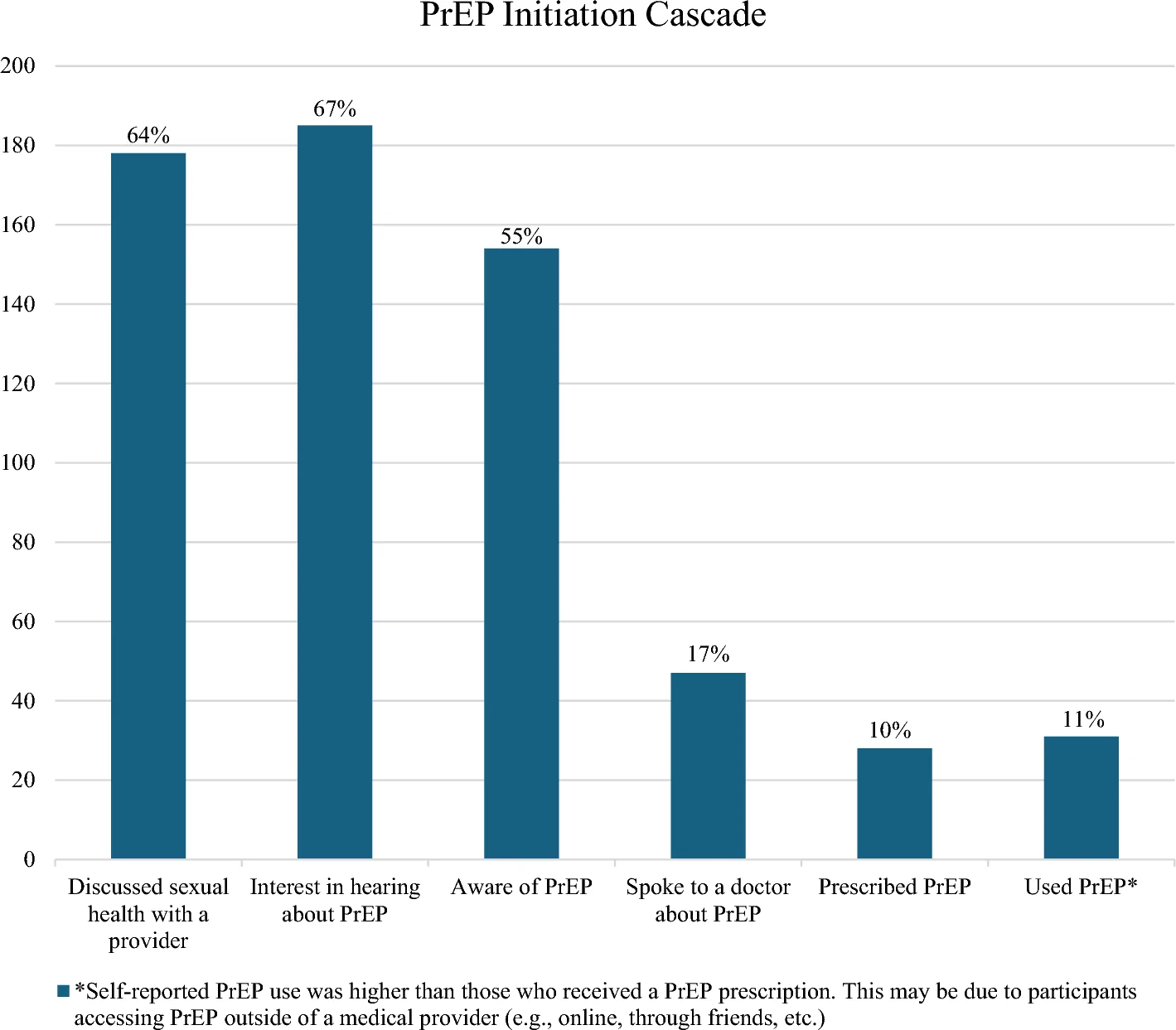
Primary care patients (n = 278) experiences with the PrEP initiation cascade

Patients had varying levels of engagement with the PrEP Initiation Cascade (Fig. 2). Most (64%) had discussed their sexual health with their provider in the last 12 months. Similarly, two-thirds (67%) of patients indicated that they wanted to hear about PrEP from their PCP. Just over half (55%) of patients were aware of PrEP, however only 17% had spoken to a provider about PrEP. Of those who had spoken to a provider about PrEP, 60% were prescribed it. In our total sample of primary care patients, only 11% had ever used PrEP.

### Discussing Sexual Health with a Provider

Overall, most (64%) participants had spoken with their provider about their sexual health in the past 12 months. In multivariable analyses (Table 2), patients who were younger [0.96 (0.94, 0.99)] were more likely to have spoken with their provider about their sexual health. Also, compared to those not diagnosed in the last 6 months, those diagnosed with an STI in the last 6 months were more likely to have discussed their sexual health with their provider [3.33 (1.73, 6.42)]. Compared to those who were never religious, those who are currently religious, but not raised religious [5.85 (1.10, 31.16)] and those who are currently religious and raised religious [2.18 (1.02, 4.66)] were more likely to have spoken with their provider about their sexual health.

**Table 2:** Multivariable logistic regression for PrEP initiation cascade among patients seen in primary care (n = 278)

	Discussing sexual health with a provider	Interest in hearing about PrEP	PrEP awareness	Speaking to a doctor about PrEP	Being prescribed PrEP **	PrEP Use
N, Percentage yes	178, 64%	185, 67%	154, 55%	47, 17%	28, 10%	31, 11%
	aOR (95% CI)	aOR (95% CI)	aOR (95% CI)	aOR (95% CI)	aOR (95% CI)	aOR (95% CI)
Age	**0.96 (0.94, 0.99)**	0.99 (0.96, 1.01)	0.98 (0.96, 1.01)	1.03 (0.98, 1.07)	1.05 (1.00, 1.11)	**1.05 (1.01, 1.10)**
Gender						
Male	Referent	Referent	Referent	Referent	Referent	Referent
Female	1.29 (0.68, 2.45)	1.26 (0.68, 2.40)	1.24 (0.65, 2.28)	0.37 (0.12, 1.09)	0.34 (0.09, 1.35)	0.13 (0.13, 1.39)
Transgender/non-binary	1.58 (0.59, 4.23)	**4.88 (1.63, 14.59)**	2.66 (0.94, 7.54)	2.66 (0.60, 11.84)	6.86 (0.90, 52.46)	2.73 (0.56, 13.36)
Race						
White	Referent	Referent	Referent	Referent	Referent	Referent
Black	0.69 (0.33, 1.43)	1.83 (0.91, 3.70)	0.86 (0.42, 1.76)	**3.29 (1.09, 9.91)**	2.21 (0.66, 7.41)	1.79 (0.60, 5.36)
Another race/ethnicity	1.12 (0.45, 2.78)	1.47 (0.62, 3.47)	1.16 (0.48, 2.80)	2.46 (0.68, 8.97)	1.00 (0.22, 4.49)	0.80 (0.19, 3.27)
Sexual orientation						
Heterosexual/straight	Referent	Referent	Referent	Referent	Referent	Referent
Homosexual/gay/lesbian	2.51 (0.84, 7.46)	**3.38 (1.07, 10.69)**	**7.70 (2.28, 26.03)**	**10.29 (3.00, 35.30)**	3.87 (0.95, 15.81)	2.60 (0.73, 9.31)
Bisexual	1.37 (0.49, 3.79)	1.45 (0.54, 3.93)	**3.67 (1.24, 10.86)**	**19.29 (5.38, 69.20)**	**9.56 (2.15, 42.50)**	**5.52 (1.43, 21.24)**
Other/prefer not to disclose	1.13 (0.36, 3.58)	1.39 (0.54, 5.31)	2.44 (0.79, 7.58)	3.02 (0.65, 14.00)	3.71 (0.64, 21.53)	3.51 (0.76, 16.22)
Marital status						
Married	Referent	Referent	Referent	Referent	Referent	Referent
Divorced/separated/widowed	0.96 (0.32, 2.87)	1.22 (0.40, 3.72)	2.20 (0.76, 6.42)	0.66 (0.22, 3.90)	0.15 (0.01, 2.21)	0.62 (0.07, 5.23)
Single	0.69 (0.32, 1.50)	0.78 (0.36, 1.67)	1.86 (0.87, 3.98)	1.32 (0.34, 5.19)	1.49 (0.31, 7.09)	2.52 (0.54, 11.84)
Education						
Less than high school diploma	Referent	Referent	Referent	Referent	Referent	Referent
High school diploma	0.43 (0.10, 1.93)	2.05 (0.56, 7.42)	0.61 (0.16, 2.28)	0.43 (0.06, 3.00)	*	4.80 (0.43, 54.05)
Some college but less than a bachelor’s degree	1.17 (0.25, 5.37)	1.77 (0.49, 6.40)	1.59 (0.42, 5.99)	0.95 (0.14, 6.62)	*	4.11 (0.33, 51.50)
Bachelor’s degree	0.90 (0.21, 3.85)	2.14 (0.63, 7.24)	1.26 (0.36, 4.39)	0.42 (0.07, 2.71)	*	4.58 (0.42, 49.39)
Master’s degree or more	0.31 (0.07, 1.48)	**5.14 (1.26, 21.05)**	1.93 (0.48, 7.75)	0.42 (0.05, 3.37)	*	3.34 (0.26, 43.22)
Religiosity						
Never religious	Referent	Referent	Referent	Referent	Referent	Referent
Raised religious, not currently religious	1.05 (0.53, 2.08)	**2.04 (1.04, 4.02)**	**2.42 (1.20, 4.88)**	0.96 (0.34, 2.73)	1.04 (0.33, 3.26)	1.11 (0.37, 3.32)
Currently religious, not raised religious	**5.85 (1.10, 31.16)**	2.12 (0.54, 8.29)	2.53 (0.67, 9.49)	1.72 (0.19, 15.35)	0.62 (0.05, 8.08)	1.89 (0.23, 15.23)
Raised and currently religious	**2.18 (1.02, 4.66)**	**2.48 (1.20, 5.12)**	**2.39 (1.15, 4.96)**	1.45 (0.42, 4.95)	0.57 (0.13, 2.39)	0.52 (0.13, 2.09)
Sexually transmitted infections						
Not diagnosed in last 6 months	Referent	Referent	Referent	Referent	Referent	Referent
Diagnosed in last 6 months	**3.33 (1.73, 6.42)**	1.60 (0.87, 2.95)	1.55 (0.85, 2.84)	**7.49 (2.31, 24.24)**	**19·29 (3.17, 117.20)**	**8.85 (2.30, 34.09)**

^*^Omitted due to small cell size

^**^10% of population had ever been prescribed PrEP, of the people who had spoken to a doctor about PrEP 60% had been prescribed it

### Interest in Hearing About PrEP

Most participants (67%) reported they wanted their provider to tell them about a preventive medication for HIV. In multivariable analyses, compared to males, transgender/non-binary patients were more likely to want to hear about PrEP from their provider [4.88 (1.63, 14.59)]. Participants who identified as homosexual/gay/lesbian were more likely to want to hear about PrEP from their provider [3.38 (1.07, 10.69)] than participants who identified as heterosexual. Compared to participants with less than a high school diploma, participants with a Master’s degree or more [5.14 (1.26, 21.05)] were more likely to want to hear about PrEP from their provider. Compared to participants who were never religious, participants who were raised religious but not currently religious [2.04 (1.04, 4.02)] and those who were raised religious and currently religious [2.48 (1.20, 5.12)] were more likely to want to hear about PrEP from their provider.

### PrEP Awareness

Just over half (55%) of participants had ever heard of PrEP. In multivariable analyses, compared to individuals who identified as heterosexual/straight, participants who identified as homosexual/gay/lesbian [7.70 (2.28, 26.03)] or bisexual [3.67 (1.24, 10.86)] were more likely to have ever heard of PrEP. Compared to participants who have never been religious, those who were raised religious and were not currently religious [2.42 (1.20, 4.88)] and those who were raised religious and were currently religious [2.39 (1.15, 4.96)] were more likely to have ever heard of PrEP.

## Speaking to a Doctor About PrEP

Fewer than one in five (17%) of participants had ever spoken to a doctor about PrEP. In multivariable analyses, compared to White participants, Black participants were more likely to have spoken with a doctor about PrEP [3.29 (1.09, 9.91)]. Participants who identified as homosexual/gay/lesbian [10.29 (3.00, 35.30)] or bisexual [19.29 (5.38, 69.20)] were more likely to have spoken with a doctor about PrEP compared to participants who identified as heterosexual/straight. Participants who were diagnosed with an STI in the last 6 months were more likely to have spoken to a doctor about PrEP [7.49 (2.31, 24.24)] compared to those who had not been diagnosed with an STI in the last 6 months.

### Being Prescribed PrEP

Only 10% of the entire sample had ever been prescribed PrEP. When examining those who had spoken to a doctor about PrEP, almost two-thirds (28/47, 60%) had been prescribed PrEP. In multivariable analyses, participants who identified as bisexual [9.56 (2.15, 42.50)] were more likely to have been prescribed PrEP compared to participants who identified as heterosexual/straight. Additionally, participants who had been diagnosed with an STI [19.29 (3.17, 117.20)] were more likely to have ever been prescribed PrEP compared to those who had not been diagnosed with an STI in the past 6 months.

### PrEP Use

Only 11% of the total sample had ever used PrEP. In multivariable analyses, participants who were older were more likely to have ever used PrEP [1.05 (1.01, 1.10)]. Participants who identified as bisexual were more likely to have ever used PrEP [5.52 (1.43, 21.24)] compared to those who identified as heterosexual/straight and those who had been diagnosed with an STI in the past 6 months were more likely to have ever used PrEP [8.85 (2.30, 34.09)] compared to those who had not been diagnosed with an STI in the last 6 months.

## Discussion

In this study, we built upon the PrEP Care Continuum [16] to examine additional steps prior to PrEP initiation among a sample of primary care patients in a large health system. We call this the PrEP Initiation Cascade. We propose eight steps that are important to understand initiating a patient on PrEP: (1) discussing sexual health with a provider, (2) interest in hearing about PrEP, (3) PrEP awareness, (4) PrEP willingness, (5) PrEP intentions, (6) speaking with a provider about PrEP, (7) being prescribed PrEP, (8) using PrEP. PrEP awareness, prescription, and use are included in the PrEP Care Continuum; steps 1, 2, and 4–6 are additional steps to PrEP initiation. We found that most participants discussed their sexual health with, and wanted to hear about PrEP from their provider; yet, less than 20% had spoken with their provider about PrEP. Slightly over half of all participants had ever heard of PrEP and only one in 10 had ever used PrEP. In multivariable analyses, we found that participants were more likely to have spoken with a doctor about PrEP or been prescribed or used PrEP based on race, gender, and sexual orientation. Religiosity was significantly associated with having spoken with a provider about sexual health, wanting to hear about and having ever heard about PrEP.

The PrEP Care Continuum defines nine steps regarding PrEP [16]. Even before Step One of the PrEP Care Continuum, “identifying individuals at highest risk for contracting HIV”, we need to normalize discussing sexual health and PrEP. We asked patients if they had discussed their sexual health and if they had spoken about PrEP with a provider. PCPs may be a good source of information sharing to the general population; however, acknowledging patients’ communication preferences and autonomy are paramount. PCPs face constraints to prescribing PrEP, such as lower knowledge of PrEP compared to HIV specialists [6], limited time and discomfort discussing sexual health [19]. Multi-level interventions [20] to address both patient and provider barriers to PrEP initiation may be necessary, as providers may need additional training on discussing sexual health and PrEP with patients and changes to their workflow to have time for these discussions. The PrEP Care Continuum also includes “facilitating PrEP access,” which includes obtaining an appointment with a provider and having a way to pay for PrEP (e.g., insurance or medication assistance programs). However, we believe a more nuanced understanding of “facilitating access”, particularly from the patient perspective, is necessary.

We propose understanding if patients want to hear about PrEP from their provider, whether they are willing to use and intend to use PrEP. We asked participants about their interest in taking PrEP, but did not ask about their willingness or intent to take PrEP. There may be nuanced differences between how people respond to interest, willingness, and intentions to use PrEP. Studies have shown there are varying rates of willingness to take PrEP based on priority population, ranging from 46–74% [21, 22]. However, there are differences in who is hypothetically willing to use PrEP and those who intend to take PrEP [23]. We did not ask about PrEP willingness nor intentions; these are conceptual placeholders for additional research. Future research using the PrEP Initiation Cascade should include willingness and intentions to use PrEP despite experienced barriers to PrEP initiation.

We found that participants who reported being currently religious were significantly more likely to have discussed their sexual health with their provider, want to hear about PrEP from their provider, and to have ever heard of PrEP compared to people who were never religious. This finding persists for participants who were not raised religious as well as raised religious. Being religious may impact with whom people are comfortable or open to discussing sex [24]. People who are religious may not feel comfortable discussing their sexual health with anyone else besides their medical provider, highlighting the protective role providers may have when discussing sexual health with patients. This finding is contrary to research published by Garcia et. al. [25], who found current and past experiences with religious institutions were related to men’s mistrust of medications, such as PrEP, and medical institutions. Being religious may positively or negatively impact patient experiences with PrEP initiation. Future research should continue to explore the role religion plays in willingness to discuss sexual health and PrEP between patients and providers.

We also found that people who were raised religious were more likely to want to hear about PrEP from a provider and to have ever heard of PrEP. This may suggest that people who were raised religious trust their provider and want their provider to be a source of information on preventive medications like PrEP. Faith leaders are crucial influencers in HIV prevention messaging, especially among Black/African Americans in the U.S [26]. Our findings align with prior research suggesting that individuals who identify as Black who were currently religious were more likely to be willing to use PrEP [27]. However, most research into the role of religion in HIV prevention has occurred in the Deep South [28] or among Black individuals [26]. PrEP initiation may be impacted by religion or geographic area due to stigma, beliefs about sex, and sexual health prevention. Our research is the first known study to examine how religion impacts PrEP initiation in a primary care setting among diverse patients in the Midwest. Future research should continue to examine how and why previous religious experiences influence HIV prevention among other populations or geographic regions.

In our sample of primary care patients, we found that sexual orientation impacted PrEP initiation. Gay patients were more likely to have spoken with their provider about PrEP, while bisexual patients were more likely to have spoken with their provider about PrEP, been prescribed PrEP, and ever used PrEP compared to individuals who identify as heterosexual/straight. Sexual minority men believe their providers have an ethical responsibility to initiate PrEP conversations, however providers perceive these conversations to be optional [29]. Few studies have examined PrEP engagement exclusively among individuals who identify as bisexual [30]. PCPs think sexual minority men should be prescribed PrEP [31] but are less likely to prescribe PrEP compared to HIV providers [6]. PCPs may need additional support via PrEP trainings or ways to identify patients who may benefit from PrEP to increase their prescribing practices. Future research should train providers how to speak with patients who may be at increased risk of acquiring HIV and efficiently identify patients who may benefit from PrEP.

We found only about half of all participants who discussed PrEP with their provider were prescribed PrEP. Consistent with the literature [32]. we found that Black individuals were not significantly more likely than White individuals to have ever been prescribed or ever used PrEP, even though HIV incidence is higher among Black. Future research should examine methods to meet the needs of Black patients and address provider biases with regards to prescribing PrEP. This may include understanding how intersectional identities (e.g., Black and gay identities) influence conversations with providers and decision-making regarding PrEP.

This study has many strengths. We built upon the PrEP Care Continuum to define the PrEP Initiation Cascade – a crucial step in understanding where patients engage with and disengage from PrEP initiation. We surveyed patients who were seen in primary care in a large Midwest health system. We used robust sampling to ensure that at least half of participants met PrEP indications, including STI diagnoses, and that race and age distributions of participants were similar to people who were diagnosed with HIV in the same geographic area. We identified demographic characteristics, including religion, that were associated with each step of the PrEP Initiation Cascade. However, there are limitations to this study. First, we surveyed primary care patients from one health system; despite this study taking place in a large and diverse health system, patient experiences with PrEP in other health systems may be different. Additionally, the survey focused on patient self-reported experiences with PrEP initiation. It is possible that their actual experiences differed from what was reported in this survey. Second, we recruited patients who had a primary care appointment within the past 6 months. Patients not seen recently by a provider may have more difficulty initiating PrEP and may disengage with the PrEP Initiation Cascade at different time points and thus may need different supports to initiate PrEP. Third, some of cascade components may not necessarily occur sequentially (i.e., discussed sexual health with provider and interest in hearing about PrEP), however both are needed to initiate PrEP. Finally, we did not account for the patient’s provider in these analyses, as the purpose of this study was to understand patient experiences with PrEP initiation. There may be certain providers who are comfortable prescribing PrEP and we do not know if their patients are overrepresented in these analyses. Conversely, we may be overrepresenting patients of providers who are not comfortable prescribing PrEP in these analyses.

## Conclusions

We defined eight steps of the PrEP Initiation Cascade: (1) discussing sexual health with a provider, (2) interest in hearing about PrEP, (3) PrEP awareness, (4) PrEP willingness, (5) PrEP intentions, (6) speaking with a provider about PrEP, (7) being prescribed PrEP, (8) PrEP use. We found primary care patients have varied engagement with the PrEP Initiation Cascade. Religion and demographic characteristics influence engagement with different steps. To make strides towards ending the HIV epidemic, we need to understand when patients disengage with the PrEP Initiation Cascade and train PCPs to keep those at greatest risk of HIV engaged in the PrEP Initiation Cascade. Understanding when people disengage with the PrEP Initiation Cascade may be the missing link to understanding how and when to support a broad proportion of the patient population with PrEP initiation.

## Supplementary Information

Below is the link to the electronic supplementary material.

Supplementary file1 (DOCX 17 KB)
