# Supplementary material for: The HIV Pre-exposure Prophylaxis (PrEP) Initiation Cascade: Patient Experiences with Primary Care in a Health Care System Before Starting PrEP
Source: AIDS Behav. 2026 Apr 28;30(11):3544–53. doi: 10.1007/s10461-026-05137-y (PMC13294956; doi:10.1007/s10461-026-05137-y)
Supplement: Supplementary file 1 [file 10461_2026_5137_MOESM1_ESM.docx]

**Supplemental Table**

|  | **Full Analytic Sample (n=278)** | **Participants with STI diagnosis in last 6 months (n=113)** | **Participants without an STI diagnosis in last 6 months (n=165)** | **P-value comparing participants with and without an STI diagnosis in the last 6 months** |
| --- | --- | --- | --- | --- |
| Age (M, SD) | 35.2 (12.8) | 34.3 (11.9) | 35.9 (13.4) | 0.48 |
| **Gender** | | | |  |
| Male | 112 (40.3) | 57 (50.4) | 55 (44) | **<0.001** |
| Female | 124 (44.6) | 54 (47.8) | 70 (56) |  |
| Transgender/Non-Binary | 42 (15.1) | 2 (1.8) | 40 (24.2) |  |
| **Race** | | | |  |
| White | 70 (25.2) | 25 (22.1) | 45 (27.3) | 0.60 |
| Black | 161 (57.9) | 69 (61.1) | 92 (55.8) |  |
| Another Race/Ethnicity | 47 (16.9) | 19 (16.8) | 28 (17.0) |  |
| **Sexual Orientation** | | | |  |
| Heterosexual/Straight | 63 (22.7) | 67 (59.3) | 123 (74.5) | **<0.001** |
| Homosexual/Gay/Lesbian | 27 (9.7) | 24 (21.2) | 9 (5.5) |  |
| Bisexual | 188 (67.6) | 13 (11.5) | 19 (11.5) |  |
| Other/Prefer Not to Disclose | 63 (22.7) | 9 (8.0) | 14 (8.5) |  |
| **Marital Status** | | | |  |
| Married | 63 (22.7) | 17 (15.0) | 46 (27.9) | **0.04** |
| Divorced/Separated/Widowed | 27 (9.7) | 12 (10.6) | 15 (9.1) |  |
| Single | 188 (67.6) | 84 (74.3) | 104 (63.0) |  |
| **Education** | | | |  |
| Less than High School Diploma | 15 (5.4) | 10 (8.8) | 5 (3.0) | 0.19 |
| High School Diploma | 55 (19.8) | 23 (20.4) | 32 (19.4) |  |
| Some college but less than a Bachelor’s Degree | 56 (20.1) | 21 (18.6) | 35 (21.2) |  |
| Bachelor’s Degree | 110 (39.6) | 46 (40.7) | 64 (38.8) |  |
| Master’s Degree or More | 42 (15.1) | 13 (11.5) | 29 (17.6) |  |
| **Religiosity** | | | |  |
| Never Religious | 99 (35.6) | 41 (36.3) | 58 (35.2) | 0.97 |
| Raised Religious, Not Currently Religious | 93 (33.5) | 39 (34.5) | 54 (32.7) |  |
| Currently Religious, Not Raised Religious | 13 (4.7) | 5 (4.4) | 8 (4.8) |  |
| Raised and Currently Religious | 73 (26.3) | 28 (24.8) | 45 (27.3) |  |
